# Multimodality Imaging Assessment of Ocular Ischemic Syndrome

**DOI:** 10.1155/2017/4169135

**Published:** 2017-12-10

**Authors:** Hui Wang, Yanling Wang, Hongyang Li

**Affiliations:** Department of Ophthalmology, Beijing Friendship Hospital, Capital Medical University, Beijing 100050, China

## Abstract

**Objectives:**

To assess the underlying mechanisms of OIS and confirm the haemodynamic and retinal structure changes of early OIS.

**Methods:**

An observational cross-sectional study was conducted of 60 internal carotid artery (ICA) stenosis patients, and they were divided into OIS and control group. Colour doppler imaging, optical coherence tomography, and fundus fluorescein angiography were performed.

**Results:**

The middle cerebral artery (MCA) stenosis differs significantly between the two groups. More OIS patients had new collateral patency of posterior communicating artery (PCoA) and retrograde flow via the ophthalmic artery (OA) (*p* < 0.001). The peak systolic velocity (PSV) in central retinal artery (CRA) and choroidal thickness (CT) was significantly reduced in OIS patients (*p* = 0.001 and *p* < 0.001). The arm-retina time (ART) and the retinal arteriovenous passage time (AVP) were prolonged in OIS patients (*p* < 0.001 and *p* = 0.001). CT, ART, and PSV of the CRA showed high sensitivity, while ART and ICA stenosis grade showed high specificity for the diagnosis of OIS according to ROC curve.

**Conclusions:**

Patients who suffered from severe ipsilateral ICA stenosis, new collateral patency of PCoAs, and MCA stenosis may be more susceptible to OIS. The most sensitive sign is PSV of CRA and CT, and the most specific sign is ART.

## 1. Introduction

Ocular ischaemia syndrome (OIS) is a potentially blinding disorder which results from significant internal carotid artery occlusion (ICAO) that arises in the context of chronic arterial hypoperfusion of the eye [1, 2]. The incidence of eye symptoms is increased when the degree of carotid artery stenosis is greater than 50%, as suggested by Lawrence and Oderich [3]. Although the incidence of OIS is not high, failure to recognise and appropriately treat this syndrome can result in serious consequences [4, 5] and the 5-year mortality rate is as high as 40% [6]. It is worth mentioning that OIS may represent the first signs of life-threatening carotid artery stenosis including cerebral transient ischaemic attacks (TIA), stroke, or myocardial infarction [7].

Colour Doppler imaging (CDI) is a noninvasive tool for the study of retrobulbar haemodynamics in OIS [8, 9]. Fundus fluorescein angiography (FFA) and indocyanine green (ICG) angiography allow for better evaluation of the retinal and choroidal vascular abnormalities in eyes with OIS [10, 11]. Ultra-wide-field angiography (UWFA) shows the wide-angle imaging of the retina, which cannot be observed using conventional FFA [12]. Those above examinations used for haemodynamic examination and the research on retinal choroid structural changes of OIS patients are limited.

The current limitations of OIS are that it can be misdiagnosed as other retinal diseases, and the best bailout time can be missed because doctors only focus on eye symptoms and do not assess the extent of intracranial ischaemia. Not all patients with stenosis, or occlusion of the internal carotid artery (ICA), can develop OIS. Patients with ICAO are at risk of future cerebral ischaemic events that may at least in part be caused by haemodynamic factors. There is a question as to why some patients diagnosed with ICA stenosis have associated OIS, while others do not.

Other questions that still need to be answered are what circumstances can easily lead to OIS when all the patients have stenosis or occlusion of the ICA and does the obstacle of the circle of Willis (CoW) plays an important role in the progression of OIS. The relationship between the CoW and OIS is still poorly understood. To confirm that brain haemodynamic changes are risk factors for early OIS development and to study the choroid structural changes in OIS, the following hypothesis was proposed: When ICAO occurs in patients with blood circulatory disorder in the CoW, the blood flow in the ophthalmic artery (OA) will reverse with the establishment of plentiful collateral circulation, and OIS may ensue. We performed a study to explain the underlying mechanisms of OIS and elucidate the relationship between CoW disorder and OIS occurrence. It is important to find an effective, highly-sensitive, multimodal detection method for OIS. Such a method can help in minimising patient blindness and improving patients' quality of life.

## 2. Methods

### 2.1. Study Design and Participants

60 ICAO and ICA stenosis patients (ipsilateral ICA stenosis ≥ 70%) were recruited from the Beijing Friendship Hospital, Capital Medical University, and an observational cross-sectional study was performed. 30 patients had ocular ischaemic symptoms (the OIS group), and the other 30 patients had no ocular ischaemic symptoms (the control group). Recruitment was carried out from May 2015 to December 2016. The authors had access to information that could identify individual participants during or after data collection.

Exclusion criteria were as follows: evidence of vitreoretinal diseases, such as age-related macular degeneration, polypoidal choroidal vasculopathy (PCV), ischaemic central retinal vein occlusion (CRVO), or diabetic retinopathy; retinal vascular lesions caused by Vogt-Koyanagi-Harada disease (VKH), Behcet's disease (BD), and rheumatoid disease; evidence of optic neuropathy, such as optic neuritis, anterior ischemic optic neuropathy, and hereditary optic neuropathy; keratopathy and severe opacity of lens; medical history of fundus laser; and intracranial injection of anti-VEGF drug. Individuals with a spherical equivalent (SE) ≥ 3D and astigmatism ≥ 2D were also excluded.

### 2.2. Examinations

#### 2.2.1. Questionnaire and Regular Ophthalmic Examination

A questionnaire was designed for face-to-face interviews, and this was used to collect the demographic data and medical history. Patients were asked about clinical presentations, such as ocular discomfort and pain. Regular ophthalmic examinations including dand slit-lamp microscopy were examined in the present study. All examinations were conducted independently but side-by-side by at least two ophthalmologists (WW and LMM) who were blind to all clinical information.

#### 2.2.2. Cephalic and Cervical Computed Tomography Angiography (CTA)

In a multidetector CTA examination, a CT scan with 64 detectors was used (Somatom Sensation; Siemens, Erlangen, Germany). Application of contrast was performed by giving intravenous (4-5 ml/s) 80 ml nonionic contrast material iopromide (Ultravist 370; Bayer Group, Berlin, Germany). Two neuroradiologist (>15 years of work experience) interpreted the CTA images and recorded the results.

The grade of ICA stenosis and all parts of the CoW, including the anterior cerebral artery (ACA), posterior cerebral artery (PCA), middle cerebral artery (MCA), anterior communicating artery (ACoA), and posterior communicating artery (PCoA), were measured. Arterial segments (ACA, PCA, and MCA) with a diameter less than normal were considered to represent stenosis. The new collateral patency of ACoA and PCoA was recorded.

#### 2.2.3. Colour Doppler Imaging (CDI)

**Figure 1:**
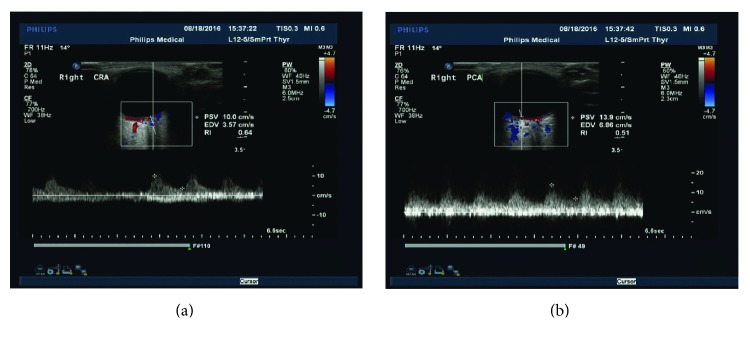
Ultrasound Doppler examinations of major ocular vessels and the measurement process of major vascular parameters. The parameters including peak systolic velocity (PSV) and end diastolic velocity (EDV). (a) The measurement process of the central retinal artery (CRA). (b) The measurement process of the posterior ciliary artery (PCA).

To evaluate the ocular circulation, we performed colour Doppler imaging (CDI) using a Philips iU22 instrument (Philips, Netherlands) and a 7.5 MHz transducer with detection limits of 0.2 mm for the vessel diameter and 1 cm/s for blood flow velocity. Peak systolic velocity (PSV) and the end diastolic velocity (EDV) were measured in the central retinal artery (CRA) and posterior ciliary artery (PCA). Reversed and antegrade blood flow was performed in the OA (Figure 1).

#### 2.2.4. Optical Coherence Tomography Examinations

**Figure 2:**
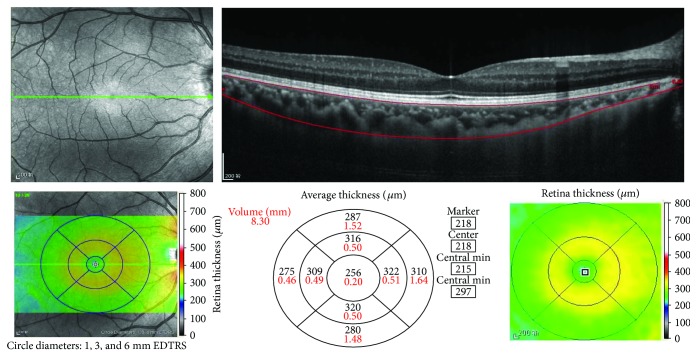
Retinal, choroidal thickness, and volume measurement. The retinal thickness and volume data can be automatically calculated and displayed within the ETDRS grid. For choroidal thickness and volume measurement, we manually moved the automatically segmented internal limiting membrane line to the corneoscleral junction. Once we changed the automatically segmented line, the choroidal thickness and volume data were automatically calculated and displayed within the ETDRS grid.

Optical coherence tomography (OCT) examinations were performed using the enhanced depth imaging (EDI) and optical coherence tomography (OCT) (Spectralis; Heidelberg Engineering, Heidelberg, Germany). A peripapillary circle scan (3.4 mm) and macular raster scan (6 mm^∗^6 mm) were used to obtain macular inner layer measurements, including the retinal nerve fiber layer (RNFL) and retinal inner layer and retinal outer layer thicknesses. The macular area was divided into three concentric circles: a central region with 1 mm diameter, an inner area with >1 mm but ≤3 mm in diameter, and an outer ring area with >3 mm but ≤6 mm in diameter. The choroidal thickness was segmented into four quadrants (inner/outer superior, inner/outer inferior, inner/outer nasal, and inner/outer temporal) (Figure 2).

#### 2.2.5. Fundus Fluorescein Angiography (FFA)

FFA was taken subsequent to injection of 5 ml of 20% sodium fluorescein intravenously. FFAs were graded independently but side-by-side by at least two ophthalmologists (WW and LMM) who were blind to all clinical information. Eyes in which media opacities prevented adequate visualisation of the fundus for fluorescein grading were excluded. The FFA indicators included choroidal filling time, the arm-retina time (ART), the retina circulation time (ACT), the retinal arteriovenous passage time (AVP), and some fundus lesion characteristics (retinal capillary nonperfusion, late leakages, and microaneurysms).

### 2.3. Positive Judgement

The diagnostic significances of the choroidal thickness, CDI, ART, AVP, and ICA stenosis grade for OIS were evaluated using the receiver operating characteristic (ROC) curve. The positive judgement value was selected from the best point as the highest cutoff point of the Youden index (*J* = sensitivity + specificity − 1) for a single indicator.

### 2.4. Statistical Analysis

The Kolmogorov-Smirnov test was used to identify the normality of distribution. The categorical data were analysed using the Fisher's exact test of chi-square tests. The independent *t*-test and Mann–Whitney *U* test were used to compare other parameters between groups. The diagnostic significances for OIS were evaluated using the ROC curve. All reported *p* values were two-sided. A *p* value <0.05 was considered statistically significant. Statistical analysis was performed using the IBM SPSS software version 21.

### 2.5. Ethical Considerations

All procedures performed in studies involving human participants were in accordance with the ethical standards of the local ethics committee of Beijing Friendship Hospital and with the 2013 Helsinki Declaration and the guideline of the International Conference on Harmonisation of Good Clinical Practice and the applicable Chinese laws. In addition, all participants provided written informed consent.

## 3. Results

### 3.1. Patient Demographics

Table 1 summarises the demographics of patients. Our cohort included 30 patients with OIS and 30 patients with only severe ICA stenosis. The incidence of severe ipsilateral ICA stenosis showed significant differences between the two groups (88.31 ± 8.25%, 77.38 ± 8.06%; *p* < 0.001).

Patients with OIS and controls showed no significant differences in age, gender, body mass index (BMI), or medical histories of hypertension or hyperlipidaemia. In addition, there were no significant differences between the two groups in terms of blood indicators (Table 1).

### 3.2. Symptoms and Ocular Manifestations

Table 2 shows the clinical presentation of the two groups. We found a decrease in best-corrected visual acuity (BCVA), vision loss, ocular pain, intraocular pressure (IOP), and visual field defect in OIS eyes compared to the control group (*p* = 0.02, *p* < 0.001).

The frequency of anterior and posterior segment signs in OIS patients was significantly greater than that in the control group (22 versus 3, 24 versus 10; *p* < 0.001 and *p* = 0.001, resp.) as shown in Table 2.

## 4. Cephalic and Cervical Computed Tomography Angiography (CTA) and Ocular Circulation Results

Table 3 showed the cephalic and cervical CTA and ocular circulation results in the OIS and control groups. The frequency of ICAO and the percentage of ICA stenosis were significantly higher in patients with OIS compared to controls (25 versus 3, 88.31 ± 8.25 versus 77.38 ± 8.06; *p* < 0.001). The artery stenosis distribution in the MCA also differed significantly between the OIS and control groups (19 versus 7; *p* = 0.004). However, there were no significant differences between the two groups in the stenosis distribution of ACA and PCA (*p* = 0.785 and *p* = 0.072, resp.). Significantly, more OIS patients had new collateral patency of the PCoA compared with the controls (21 versus 10; *p* = 0.009). However, the collateral patencies of the ACoA were not significantly different between the two groups (16 versus 17 *p* = 1.000) (Table 3).

CDI results present the flow velocities of the CRA, PCA, and antegrade and retrograde flow of the OA. The OIS patients had more antegrade flow and less retrograde flow via the OA compared to the controls (7 versus 26, 23 versus 4; *p* < 0.001, resp.). The average PSV in the CRA was 7.87 ± 1.83 m/sec in the OIS group and 9.69 ± 2.32 m/sec in the control group, while the PSV in the PCA was 7.87 ± 1.83 m/sec and 9.69 ± 2.32 m/sec (*p* = 0.001 and *p* = 0.005, resp.) (Table 3).

### 4.1. Optical Coherence Tomography (OCT) Examinations

Patients underwent OCT imaging. Average central choroidal thickness was significantly reduced in OIS patients (227.37 ± 14.46 *μ*m) relative to the control patients (245.17 ± 12.42 *μ*m; *p* < 0.001).

The retinal, RNFL, and inner retinal and outer retinal thicknesses were not significantly different between the OIS patients and controls (*p* = 0.405, *p* = 0.520, *p* = 0.514, and *p* = 0.406, resp.). In addition, there were no significant differences between the two groups in terms of the choroidal thickness (nasal quadrant, temporal quadrant, superior quadrant, or inferior quadrant) in either the inner or outer ring area (Table 4).

### 4.2. Fundus Fluorescein Angiography (FFA) Results

More OIS patients demonstrated patchy or delayed choroidal filling compared with the controls (19 versus 2; *p* < 0.001). The ART and AVP were significantly prolonged in patients with OIS compared to controls (34.33 ± 11.87 s versus 16.73 ± 4.40 s, 13.60 ± 2.94 s versus 11.37 ± 1.50 s; *p* < 0.001 and *p* = 0.001, resp.). More OIS patients exhibited various types of fundus features compared with the controls (27 versus 4; *p* < 0.001) (Table 5).

**Figure 3:**
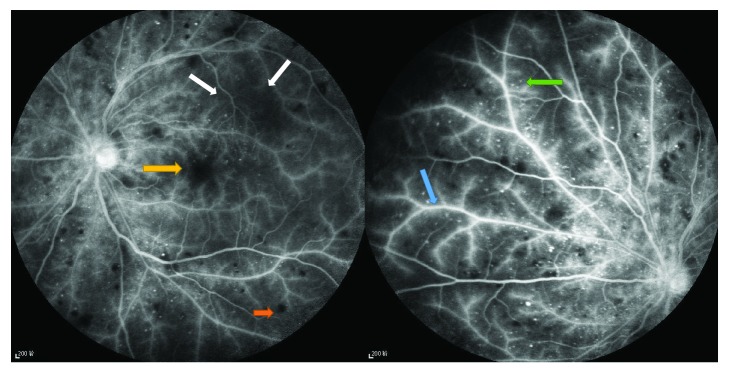
Fundus fluorescein angiogram findings of the OIS eye. The retinal veins are dilated but not tortuous. Fundus fluorescein angiogram of the eye showing staining of the vessels (blue arrow), capillary nonperfusion areas (white arrows), microaneurysms (green arrow), and hemorrhages (orange arrow). Macular edema appeared in some cases (yellow arrow).

We found some fundus lesions characteristics in OIS eyes (Figure 3).

### 4.3. Receiver Operating Characteristic (ROC) Curve Analysis

Receiver operating characteristic (ROC) curve analysis was performed for both the OIS and control groups. The ROC curve revealed that diagnostic efficiency was greatest for choroidal thickness (CT), followed by CDI, ART, AVP, and ICA stenosis grade, in that order. The positive judgement value was selected by using the optimum cutoff point of the Youden index (the maximum value of the index, *J* = sensitivity + specificity − 1) for a single indicator.

**Figure 4:**
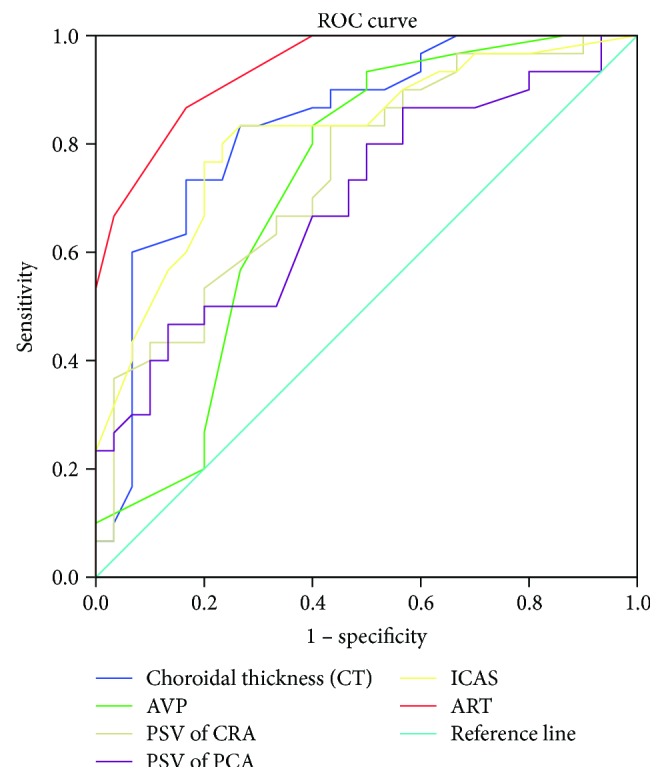
ROC curve analysis of choroidal thickness, CDI, ART, AVP, and ICA stenosis grade in OIS.

CT < 236 *μ*m, ART > 21.50 s, AVP > 12.74 s, PSV of CRA < 7.70 m/sec, PSV of PCA < 8.99 m/sec, and ICA stenosis grade > 80.65% were treated as positive. Their sensitivities for the diagnosis of OIS were 81.48%, 79.41%, 78.26%, 82.35%, 76.47%, and 75.76%, respectively. Their specificities for the diagnosis of OIS were 75.76%, 88.46%, 67.57%, 65.85%, 63.41%, and 81.48%, respectively (Figure 4).

## 5. Discussion

The present study described the ocular manifestations and clinical features of OIS and ICA stenosis patients in a Chinese population. Visual loss, orbital pain, and various anterior and posterior segment signs are the most common clinical manifestations in OIS [4, 13, 14]. Visual loss is usually related to chronic or acute retinal ischaemia and damage to the optic nerve due to secondary glaucoma [15]. Pain may be the result of increased IOP or may be ischaemic in origin [1]. The present study showed the same ocular manifestations as described in the previous study. Ocular symptoms are signs of severe stenosis of the unilateral ICA and may occur before cerebrovascular and cardiovascular complications. Early diagnosis is necessary; if this is delayed, irreversible damage may occur.

CTA may reveal various pathologies and their variations, such as carotid system stenosis, aneurysms, and venous thrombi [16]. The CoW provides several important collateral circulation paths to maintain a sufficient blood supply if a part of the circulation becomes occluded, and stenosis of any parts of it may be a signal of ischaemic vascular disease [17–19]. In the present study, more patients with MCA stenosis were found in the OIS group, showing that patients who simultaneously suffered from severe ipsilateral ICA stenosis and MCA stenosis may be more susceptible to OIS. The PCoA is the most important collateral pathway. However, this compensatory effect will be invalidated when the degree of ICA stenosis is greater than 90%. In our study, new collateral patencies of PCoAs were found in most OIS patients, indicating that the function of ipsilateral PCoA is not activated until severe stenosis of the unilateral ICA occurs. Thus, if CTA shows new collateral patency of PCoA in ICAS patients, it may be a sign of serious brain ischaemia, and OIS may easily develop. Early screening is necessary for these people, as it is associated with the highest risk of OIS when the ipsilateral ICA exhibits severe stenosis.

CDI has been used for measuring the retrobulbar blood flow in patients with occlusive carotid artery disease and confirming the presence of the “steal phenomenon” [20]. Flow reversal in the OA produces the steal phenomenon from the ocular circulation to the low pressure intracranial circuit, causing a reduction in the retrobulbar blood flow; this may be the pathogenesis of OIS. Collateral flow via the OA has often been regarded as a marker of insufficient cerebral perfusion in previous studies [21, 22]. Similarly, a retrograde blood flow via the OA was more frequent in OIS patients in our study, indicating that it also represents insufficient ocular perfusion. Mendrinos et al. [1] summarised that a reversed OA flow pattern is a highly specific indicator of ipsilateral high-grade ICA stenosis or occlusion. In our study, we also found significantly lower CRA and PCA flow velocities in patients with OIS, which contradicts some previous studies [23, 24]. To summarise, the examination of decreased retinal blood flow in the CRA and PCA had high sensitivity in the diagnosis of OIS. The retrograde of OA is a highly specific indicator for OIS. However, CDI currently has limited clinical use at present because measurements of flow velocity in the orbital vessels are poorly reproduced [25]. Prospective longitudinal studies need to confirm the prognostic value of reduced retrobulbar haemodynamics in OIS.

OCT is a useful tool for the quantitative and qualitative assessment of retinal layers. We have demonstrated that OIS eyes have a lower CT than the controls, indicating impaired choroidal circulation in patients with OIS eyes. Knowledge of the choroidal profile and thickness in eyes may help in elucidating physiological changes and evaluating chorioretinal conditions in patients with ocular ischaemic changes caused by severe ICA stenosis. OCT may be a sensitive diagnostic tool for OIS, but it is not OIS specific, as it can also be used for eyes with hypermyopia or other disorders. In addition, there are other confounding factors, such as age, axial length, refractive error, and BMI, that can affect the measurement results [26]. Thus, we need to combine clinical symptoms and other examination methods with OCT indicators when diagnosing OIS.

Fundus fluorescein angiography can help to establish a diagnosis of OIS [11]. In the present study, delayed choroidal filling appeared in 63.33% of OIS eyes, which is in accordance with Brown and Magargal's study [11]. Prolonged choroidal filling time and ART, although not sensitive, are the most specific angiographic signs of OIS described by some researchers [1, 11]. The most sensitive angiographic sign of OIS is prolonged retinal arteriovenous time, which is present in up to 95% of cases; however, this is not OIS specific, as it can also be observed in central retinal artery occlusion (CRAO) and central retinal vein occlusion (CRVO) [1]. The results in this study were the same as those in previous studies showing that the ART and AVP were significantly prolonged in OIS patients. Staining of retinal vessels, leakage due to ischaemic endothelial cell dysfunction and the increased permeability with extravasation [27], retinal capillary nonperfusion [11, 28], and microaneurysms have been determined, in past research, to be major FFA features in OIS patients, which were also manifested in this study.

The ROC curve revealed the positive judgement value in each inspection method. CT, ART, and PSV of the CRA all showed relatively high sensitivity, while ART and the ICA stenosis grade showed high specificity for the diagnosis of OIS. However, in this research, the diagnosis effect of joint multiple diagnostic methods, such as joint detection with ART and CT or ICA stenosis and CDI, was not studied. Other less commonly used diagnostic techniques include electroretinography (ERG), visual-evoked potentials (VEP), ophthalmodynamometry and ocular plethysmography, techniques which will be utilized in future research.

Early diagnosis is crucial if blindness is to be minimised and quality of life improved for OIS patients. Ophthalmologists may be the first to deal with patients suffering from carotid artery occlusion, and they should be aware of the clinical presentation of OIS. Continued improvements in the multimodal imageological examinations increase the importance of the role of ophthalmologists increasingly important in the early detection of OIS. Joint detection of OIS, using a variety of diagnostic methods, will be important to understand in the near future.

## 6. Conclusions

Patients who simultaneously suffered from severe ipsilateral ICA stenosis, new collateral patency of PCoAs, and MCA stenosis may be more susceptible to OIS. The retrograde of OA is a highly specific indicator for OIS. OCT may be a sensitive diagnostic tool for OIS because OIS eyes also had a lower choroidal thickness. The most sensitive sign of OIS is PSV of CRA and choroidal thickness, the most specific sign is ART according to ROC curve. Joint detection of a variety diagnostic methods for OIS will be very important in the future.

## Figures and Tables

**Table 1:** Epidemiologic characteristics of patients.

Disease	OIS	Control	*p* value
Number of eyes (*n*)	30	30	
Max carotid stenosis (%, mean ± SD)	88.31 ± 8.25	77.38 ± 8.06	*p* < 0.001^∗^
Age (year, mean ± SD)	64.30 ± 5.68	62.90 ± 7.35	*p* = 0.413^∗^
Gender (male/female)	19/11	17/13	*p* = 0.792^‡^
BMI (kg/m^2^)	24.43 ± 2.73	25.73 ± 3.05	*p* = 0.087^∗^
Hypertension/n%	17/13	18/12	*p* = 1.000^‡^
Hyperlipidemia/n%	16/14	15/15	*p* = 1.000^‡^
Blood glucose (mmol/L)	5.65 (5.10, 6.67)	5.93 (5.20, 8.24)	*p* = 0.433^†^
Blood lipids (mmol/L)	4.59 ± 1.21	4.42 ± 1.10	*p* = 0.587^∗^
Triglyceride (mmol/L)	1.80 (1.06,2.11)	1.54 (1.02, 2.20)	*p* = 0.690^†^

OIS: ocular ischemia syndrome; BMI: body mass index. ^∗^*p* values were calculated using the independent *t*-test. ^†^*p* values were calculated using the Mann–Whitney *U* test. ^‡^Fisher's exact test of chi-square tests.

**Table 2:** Clinical presentation of the two groups.

	OIS	Control	*p* value
Number of patients (*n*)	30	30	
Vision loss, *n* (%)	27 (90.00%)	8 (26.67%)	*p* < 0.001^‡^
Ocular pain, *n* (%)	11 (36.67%)	2 (6.67%)	*p* < 0.001^‡^
Visual field defect, *n* (%)	12 (40.00%)	0 (0.00%)	*p* < 0.001^‡^
Baseline BCVA (LogMAR)	0.30 ± 0.16	0.17 ± 0.15	*p* = 0.002^∗^
Refractive error (SE)	−0.88 (−1.56, −0.50)	−0.75 (−1.25, 0.00)	*p* = 0.490^†^
IOP (mmHg, mean ± SD)	19.41 ± 4.91	13.91 ± 2.52	*p* < 0.001^∗^
Anterior segment signs	22	3	*p* < 0.001^‡^
Corneal edema	6 (20.00%)	2 (6.67%)	*p* = 0.254^‡^
Episcleral injection	8 (26.67%)	0 (0.00%)	*p* = 0.005^‡^
Anterior uveitis (flare, cells, KP)	8 (26.67%)	0 (0.00%)	*p* = 0.005^‡^
INV	22 (73.33%)	1 (3.33%)	*p* < 0.001^‡^
NVG	10 (33.33%)	0 (0.00%)	*p* = 0.001^‡^
Uveitis	2 (6.67%)	0 (0.00%)	*p* = 0.492^‡^
Posterior segment signs	24	10	*p* = 0.001^‡^
Narrowed RA	19 (63.33%)	3 (10.00%)	*p* < 0.001^‡^
Dilated RV	19 (63.33%)	4 (13.33%)	*p* < 0.001^‡^
Retinal haemorrhages	15 (50.00%)	6 (20.00%)	*p* = 0.029^‡^
Microaneurysms	21 (70.00%)	5 (16.67%)	*p* = 0.003^‡^

BCVA: best-corrected visual acuity; SE: spherical equivalent; IOP: intraocular pressure; RA: retinal artery; RV: retinal vein; INV: iris neovascularisation; NVG: neovascular glaucoma; NVD: neovascularization of disc; NVE: neovascularization of retina elsewhere. ^∗^*p* values were calculated using the independent *t*-test. ^†^*p* values were calculated using the Mann–Whitney *U* test. ^‡^Fisher's exact test of chi-square tests.

**Table 3:** Comparison of CTA and CDI results between OIS and Control.

	OIS	Control	*p* value
Number of eyes (*n*)	30	30	
CTA results	25 (83.33%)	3 (10.10%)	*p* < 0.001^‡^
Ipsilateral ICAO, *n* (%)	25 (83.33%)	3 (10.10%)	*p* < 0.001^‡^
Ipsilateral ICAS (mean ± SD)	88.31 ± 8.25	77.38 ± 8.06	*p* < 0.001^∗^
CoW stenosis, *n* (%)			
ACA	9 (30.00%)	11 (36.67%)	*p* = 0.785^‡^
PCA	11 (36.67%)	4 (13.33%)	*p* = 0.072^‡^
MCA	19 (63.33%)	7 (23.33%)	*p* = 0.004^‡^
Collateral patency, *n* (%)			
ACoA	16 (53.33%)	17 (56.67%)	*p* = 1.000
PCoA	21 (70.00%)	10 (33.33%)	*p* = 0.009
CDI results			
OA flow direction, *n* (%)			
Antegrade	7 (23.33%)	26 (86.67%)	*p* < 0.001^‡^
Retrograde	23 (76.67%)	4 (13.33%)	*p* < 0.001^‡^
PSV (m/sec)			
CRA	7.87 ± 1.83	9.69 ± 2.32	*p* = 0.001^∗^
PCA	9.42 ± 2.31	11.39 ± 2.93	*p* = 0.005^∗^

ICAO: internal carotid artery occlusion; ICAS: internal carotid artery stenosis; CoW: the circle of Willis; ACA: anterior cerebral arteries; PCA: posterior cerebral artery; MCA: middle cerebral artery; ACoA: anterior communicating artery; PCoA: posterior communicating arteries; OA: ophthalmic artery; CRA: central retinal artery; PCA: posterior ciliary artery; PSV: peak systolic velocity. ^∗^*p* values were calculated using the independent *t*-test. ^†^*p* values were calculated using the Mann–Whitney *U* test. ^‡^Fisher's exact test of chi-square tests.

**Table 4:** Comparison of OCT between OIS and control.

	OIS	Control	*p* value
Number of eyes	30	30	
Retinal thickness(*μ*m)	266.50 ± 25.73	261.43 ± 20.75	*p* = 0.405^∗^
RNFL thickness	11.27 ± 1.93	11.57 ± 1.65	*p* = 0.520^∗^
Inner thickness	177.40 ± 23.11	173.57 ± 22.07	*p* = 0.514^∗^
Outer thickness	87.00 (83.75, 91.50)	86.50 (84.75, 89.25)	*p* = 0.406^†^
Choroidal thickness(*μ*m)			
Central choroidal thickness	227.37 ± 14.46	245.17 ± 12.42	*p* < 0.001^∗^
Inner ring area			
Nasal	316.13 ± 30.56	315.20 ± 23.92	*p* = 0.896^∗^
Temporal	302.30 ± 27.29	303.17 ± 25.72	*p* = 0.900^∗^
Superior	329.53 ± 33.31	328.83 ± 24.43	*p* = 0.926^∗^
Inferior	319.00 (305.75, 346.00)	323.00 (305.75, 346.25)	*p* = 0.847^†^
Outer ring area			
Nasal	299.97 ± 24.74	302.53 ± 19.00	*p* = 0.654^∗^
Temporal	279.00 (260.50, 286.00)	275.00 (256.00, 286.75)	*p* = 0.853^†^
Superior	292.93 ± 27.79	291.10 ± 18.28	*p* = 0.764^∗^
Inferior	282.17 ± 21.88	278.90 ± 26.95	*p* = 0.608^∗^

RNFL: retinal nerve fiber layer; mRCG: macular ganglion cell layer; mIPL: macular inner plexiform layer; mINL: macular inner nuclear layer. ^∗^*p* values were calculated using the independent *t*-test. ^†^*p* values were calculated using the Mann–Whitney *U* test. ^‡^Fisher's exact test of chi-square tests.

**Table 5:** Comparison of FFA between OIS and control.

	OIS	Control	*p* value
Number of eyes	30	30	
Circulation time (s)			
ART	34.33 ± 11.87	16.73 ± 4.40	*p* < 0.001^∗^
AVP	13.17 ± 2.23	11.37 ± 1.50	*p* = 0.001^∗^
Choroidal filling time delay, *n* (%)	19 (63.33%)	2 (6.67%)	*p* < 0.001^‡^
Fundus features	27 (90.00%)	4 (13.33%)	*p* < 0.001^‡^
Staining of the retinal vessels, *n* (%)	27 (90.00%)	2 (6.67%)	*p* < 0.001^‡^
Leakage, *n* (%)	26 (86.67%)	4 (13.33%)	*p* < 0.001^‡^
Macular oedema, *n* (%)	16 (53.33%)	1 (3.33%)	*p* < 0.001^‡^
Retinal neovascularization, *n* (%)	14 (46.67%)	2 (6.67%)	*p* < 0.001^‡^
Capillary nonperfusion area, *n* (%)	20 (66.67%)	4 (13.33%)	*p* < 0.001^‡^
Microaneurysms, *n* (%)	19 (63.33%)	2 (6.67%)	*p* < 0.001^‡^

FFA: fundus fluorescein angiography; ART: the arm-retina time; AVP: retinal arteriovenous passage time; CNP: retinal capillary nonperfusion. ^∗^*p* values were calculated using the independent *t*-test. ^†^*p* values were calculated using the Mann–Whitney *U* test. ^‡^Fisher's exact test of chi-square tests.
